# 68Ga-DOTATOC-PET/MRI—A Secure One-Stop Shop Imaging Tool for Robotic Radiosurgery Treatment Planning in Patients with Optic Nerve Sheath Meningioma

**DOI:** 10.3390/cancers13133305

**Published:** 2021-07-01

**Authors:** Josefine Graef, Christian Furth, Anne Kathrin Kluge, Gueliz Acker, Melina Kord, Zoe Zimmermann, Holger Amthauer, Marcus Makowski, Franziska Loebel, Peter Vajkoczy, Volker Budach, Carolin Senger

**Affiliations:** 1 Department of Nuclear Medicine, Charité—Universitätsmedizin Berlin, Corporate Member of Freie Universität Berlin, Humboldt-Universität zu Berlin, Berlin Institute of Health, 13353 Berlin, Germany; christian.furth@charite.de (C.F.); holger.amthauer@charite.de (H.A.); 2 Department of Radiation Oncology, Charité—Universitätsmedizin Berlin, Corporate Member of Freie Universität Berlin, Humboldt-Universität zu Berlin, Berlin Institute of Health, 13353 Berlin, Germany; anne.kluge@charite.de (A.K.K.); melina.kord@charite.de (M.K.); zoe.zimmermann@charite.de (Z.Z.); volker.budach@charite.de (V.B.); carolin.senger@charite.de (C.S.); 3 Berlin Institute of Health at Charité—Universitätsmedizin Berlin, BIH Academy, Clinician Scientist Program, 10117 Berlin, Germany; gueliz.acker@charite.de; 4 Department of Neurosurgery, Charité—Universitätsmedizin Berlin, Corporate Member of Freie Universität Berlin, Humboldt-Universität zu Berlin, Berlin Institute of Health, 10117 Berlin, Germany; franziska.loebel@charite.de (F.L.); peter.vajkoczy@charite.de (P.V.); 5 Department of Diagnostic and Interventional Radiology, School of Medicine, Technical University of Munich, 81675 Munich, Germany; marcus.makowski@tum.de

**Keywords:** 68Ga-DOTATOC, hybrid imaging, optic nerve sheath meningioma, PET/MRI, robotic radiosurgery, stereotactic radiotherapy

## Abstract

**Simple Summary:**

Through combined information about spatial spread in magnetic resonance imaging (MRI) and positron emission tomography (PET), gallium-68-labeled (DOTA0-Phe1-Tyr3) octreotide (Ga68-DOTATOC) PET/MRI before radiosurgery allows safe treatment planning for optic nerve sheath meningiomas (ONSM). Moreover, because of specific receptor stocking, diagnostic accuracy is improved without the need for biopsy. Thus, Ga68-DOTATOC-PET/MRI can be considered a safe “one-stop shop” image tool prior to radiosurgery for ONSM.

**Abstract:**

Optic nerve sheath meningiomas (ONSM) are rare but can lead to irreversible blindness. Hybrid imaging may enhance tumor delineation and diagnostic accuracy via receptor binding. However, relevant clinical data for ONSM are lacking. We evaluated the feasibility of receptor-based hybrid imaging prior to robotic radiosurgery (RRS). We retrospectively analyzed all of our institution’s patients with suspected ONSM who underwent combined positron emission tomography and magnetic resonance imaging (PET/MRI) with gallium-68-labeled (DOTA0-Phe1-Tyr3) octreotide (Ga68-DOTATOC) before RRS between 2018 and 2019. Eight patients with ten suspected ONSM (female = 7; median age, 51.2 years; IQR, 43.0–66.0) were included. Nine out of ten ONSM were deemed PET-positive with a median standard uptake value (SUV) max of 5.6 (IQR, 2.6–7.8). For all nine ONSM that presented 68Ga-DOTATOC uptake, hybrid PET/MRI was used for target volume contouring prior to RSS. At a median follow-up of 11.7 months (IQR, 9.4–16.4), tumor control was achieved in all patients. Radiosurgery resulted in the improvement of visual acuity in two of eight patients, whereas six showed stable vision. Ga68-DOTATOC-PET/MRI can be used for target volume contouring prior to RRS for ONSM as it enables safe treatment planning and improves diagnostic accuracy.

## 1. Introduction

Optic nerve sheath meningiomas (ONSM) are a rare presentation of meningiomas that may lead to severe complications due to the involvement of the optic nerve [1]. Although most patients present with slowly progressive symptoms and variable visual loss as a result of the slow tumor growth, ONSM pose a risk for irreversible blindness [2,3].

The gold standard for the diagnosis of ONSM is magnetic resonance imaging (MRI), as ONSM are distinguishable on gadolinium-enhanced and fat-suppressed T1-weighted images [4]. On axial MRI sequences, the contrast-enhancing outer layers of the ONSM on both sides and the non-enhancing inner layer of the optic nerve create the typical “tram-track” sign. On coronal images, this appears as a “doughnut” sign. Therefore, the diagnosis of ONSM is based on combined MRI findings and clinical presentation [5].

However, in some cases, the typical signs may be missing and differentiation between ONSM and other intraorbital tumors, such as optic nerve glioma, using MRI alone may be difficult. In these cases, positron emission tomography (PET) imaging with gallium-68-labeled (DOTA0-Phe1-Tyr3) octreotide (Ga68-DOTATOC) can improve the accuracy of diagnosis without the need for invasive biopsy [6,7]. Somatostatin receptor subtype 2, expressed by meningiomas, is used as a binding site for the somatostatin analog DOTATOC [8]. This receptor is found in high concentrations in meningiomas, but not in healthy brain tissue, except for the pituitary gland, and therefore it offers a very high lesion-to-background contrast [9,10,11].

In recent years, fractionated stereotactic radiotherapy has become an effective treatment option for ONSM and is the standard of care [12,13,14]. Compared to other stereotactic radiotherapies, robotic radiosurgery (RRS) delivers a precise tight dose to the tumor while sparing the perilesional tissue or structures of risk, such as the affected optic nerve or chiasm [15]. Before the irradiation dose can be applied, accurate treatment planning is necessary with delineation of the planning target volume (PTV) and the healthy optic pathway. It has already been shown that 68Ga-DOTATOC-PET images offer better PTV delineation in radiation oncology as compared with standard diagnostic computer tomography (CT) and MRI alone [16,17]. The high lesion-to-background contrast that enables improved PTV delineation is particularly beneficial for ONSM because of the limited experience of radiation oncologists may have due to the rarity of the disease.

Moreover, to ensure patient safety, radiation oncologists must be certain that the correct diagnosis is made. In this regard, useful information can potentially be provided by the hybrid imaging of 68Ga-DOTATOC-PET/MRI as a one-stop-shop imaging tool. While PET/CT has become a widely available technology, combined PET and MRI systems are still relatively rare [18]. Since 68Ga-DOTATOC-PET/MRI has only recently been used in the diagnosis of meningiomas [19], to the best of our knowledge, there are no studies on its use in the diagnosis and radiosurgery treatment planning for ONSM.

In the present study, we investigated the feasibility of 68Ga-DOTATOC-PET/MRI as a diagnostic tool for ONSM and further evaluated its usefulness for PTV contouring for RRS treatment planning.

## 2. Materials and Methods

### 2.1. Study Design

We retrospectively analyzed the records of all patients with suspected ONSM who underwent 68Ga-DOTATOC-PET/MRI before RRS between August 2018 and November 2019 at the Charité CyberKnife Center, Berlin, Germany. Only patients with suspected primary ONSM were included, while those with secondary ONSM, which grew from outside the orbit, that is, from the sphenoid wing, along the optic nerve sheath, and then intraorbitally [12], were excluded. A total of 10 ONSM in 8 patients who underwent 68Ga-DOTATOC-PET/MRI before RRS were identified. These patients were part of the series that was recently published to assess local control [20].

We collected data regarding the ONSM location, visual deficits prior to radiosurgery, and histopathology, if available, after previous surgical treatments. The feasibility of 68Ga-DOTATOC-PET/MRI for the detection of ONSM, using PET imaging indicators such as positive receptor binding by 68Ga-DOTATOC and tracer uptake value, and furthermore, MRI characteristics such as contrast enhancement and localization, were analyzed using syngo.via (VB 30, Siemens Healthcare GmbH, Erlangen, Germany). The results were cross-checked with the histopathological findings, if available.

Radiation contouring was performed with MultiPlan 4.5/Precision 2.0 (Accuray Inc., Sunnyvale, CA, USA) using co-registered 68Ga-DOTATOC-PET/MRI datasets (e.g., contrast-enhanced T1-weighted MPRAGE, attenuation-corrected PET images), and we analyzed the RRS dose-volume parameters. Indicators for an effective treatment were local tumor control (LC) based on the follow-up MRI, visual acuity outcomes, and occurrence of side-effects due to radiation. Because of the limited number of patients, data are presented descriptively, and parameters are expressed as medians and interquartile ranges (IQR). For the statistical analysis we used SPSS Statistics (version 25, IBM, Armonk, NY, USA).

### 2.2. 68 Ga-DOTATOC PET/MRI

PET/MRI scans were performed using a Siemens Biograph mMR scanner (VE11P; Siemens Healthcare GmbH, Erlangen, Germany). The PET scan was acquired in a single-bed position covering the entire skull (3D list mode acquisition). We used ordered-subset expectation maximization (3 iterations, 21 subsets; voxel matrix 344 × 344 × 127; voxel size 1.0 × 1.0 × 2.0 mm) for reconstruction of PET raw data. The vendor-provided ultrashort echo-time sequence was used for attenuation and scatter correction. Among the individually adjusted MRI sequences, all patients underwent T2-weighted turbo-spin echo (TSE), diffusion-weighted, and post-contrast 3D magnetization-prepared rapid acquisition with gradient echo (MPRAGE) imaging.

### 2.3. RRS

**Figure 1:**
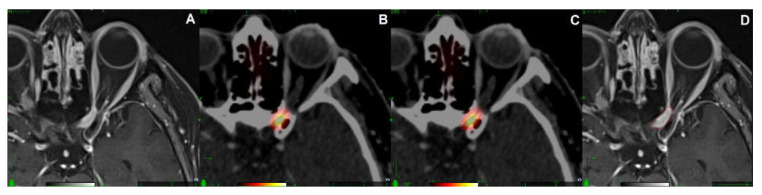
Radiosurgery contouring process: (**A**) Optic nerve sheath meningioma (ONSM) left eye difficult to see on MRI alone; (**B**) 68Ga-DOTATOC-PET/MRI co-registered with the planning CT demonstrates left canalicular location of ONSM; (**C**) initial tumor contouring based on the PET signal; (**D**) fine-tuning of the tumor volume using the MRI of the hybrid PET/MRI, no safety margin was added to generate the planning target volume (red line). MRI, magnetic resonance imaging, Ga68-DOTATOC, gallium-68-labeled (DOTA0-Phe1-Tyr3) octreotide; PET, positron emission tomography; CT, computer tomography.

Treatment planning was based on contrast-enhanced thin-slice planning CT (0.75 mm) and co-registered 68Ga-DOTATOC-PET/MRI datasets, including attenuation-corrected PET images, fat-suppressed T1-weightes sequences, T2-weighted TSE, and contrast-enhanced T1-weighted MPRAGE. Gross tumor volume (GTV) was defined as the ONSM extension based on CT and PET/MRI datasets, with a 0–1 mm safety margin added to generate the PTV, avoiding overlap with the optic nerve. The contouring process using the 68Ga-DOTATOC-PET/MRI in detail is shown in Figure 1. RRS treatment planning and dose calculation were performed using MultiPlan 4.5/Precision 2.0 (Accuray Inc., Sunnyvale, CA, USA). The ray-tracing dose calculation algorithm is routinely used. The dose was delivered using the CyberKnife Radiosurgery System VSI (Accuray Inc., Sunnyvale, CA, USA). The CyberKnife treatment technique has already been described in detail in other studies [21,22,23].

### 2.4. Follow-Up

During the follow-up period, patients were examined by MRI of the head 6 months after RRS and annually thereafter, for the current status of the treated ONSM. The pre- and post-therapeutic visual acuity was evaluated as required by the European Norm (EN ISO 8596) by the patients’ ophthalmologists.

### 2.5. Literature Review

Literature research focusing on hybrid imaging in meningiomas was performed in the PubMed database on 6–8 May 2020. The search for articles on the use of PET/MRI in ONSM showed no results; therefore, studies based on PET/MRI in meningiomas and PET imaging in ONSM were evaluated. The following free text search terms were used: “68Ga PET/MRI meningioma”, “DOTATOC PET/MRI meningioma”, “68Ga PET/CT optic nerve sheath meningioma”, and “68Ga PET/CT ONSM”. Data on ONSM imaging with PET/CT and meningioma imaging with somatostatin analogue PET/MRI were limited; therefore, all studies were included in the literature review. The characteristics, methods, and results of the studies, as well as their limitations, were summarized.

## 3. Results

### 3.1. Patients’ Characteristics

In total, eight patients with ten suspected ONSM (female = 7; median age, 51.2 years, IQR, 43.0–66.0) were included. ONSM were located in the orbital and canalicular (four patients), orbital only (three patients), or canalicular only regions (one patient). Two patients presented with bilateral ONSM. Except for one, all lesions were circular around the optic nerve. Accompanying symptoms included motility restriction (three patients), exophthalmos (two patients), and field of view restrictions (seven patients with eight meningiomas: partial restriction, *n* = 4; light-dark seeing, *n* = 4). The time between initial diagnosis and RRS was highly divergent with a median of 8.2 months (IQR, 2.8–46.4). Three patients had already undergone microsurgical resection before RRS. The time interval between surgery and RRS ranged from 2.0 to 6.0 months.

### 3.2. 68 Ga-DOTATOC-PET/MRI

**Figure 2:**
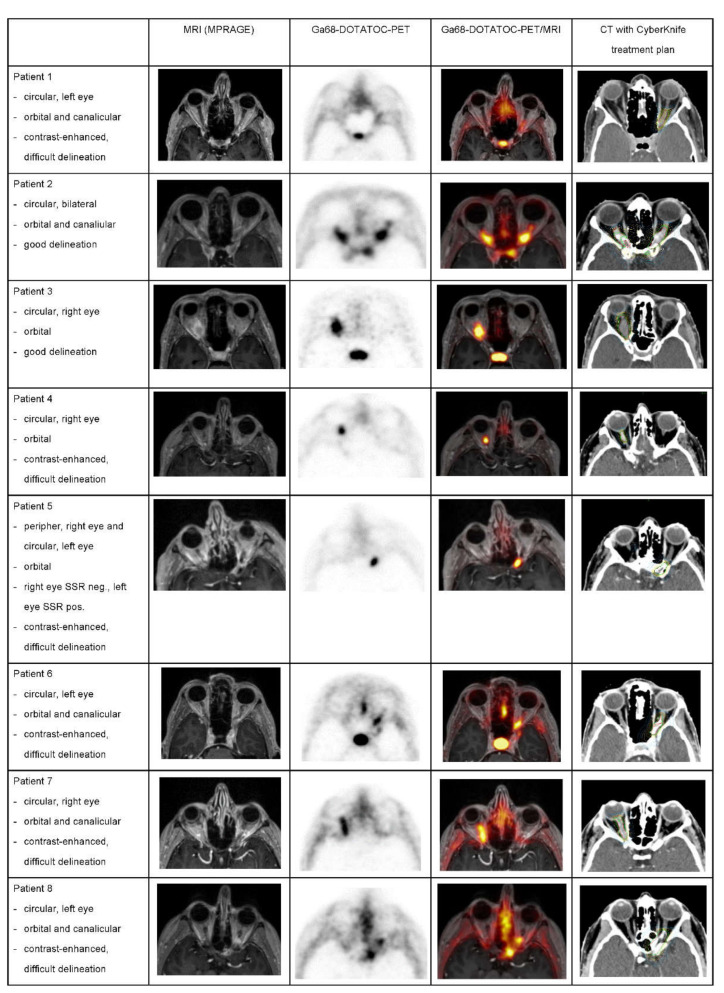
Optic nerve sheath meningiomas (ONSM) on MRI and PET images in all patients and according to the treatment plan. In patients with bilateral ONSM, only one side is presented. MRI, magnetic resonance imaging, PET, positron emission tomography; MPRAGE, contrast-enhanced T1-weighted magnetization-prepared rapid acquisition with gradient echo; Ga68-DOTATOC, gallium-68-labeled (DOTA0-Phe1-Tyr3) octreotide; SSR, somatostatin receptor; Neg, negative; Pos, positive.

First, we analyzed the feasibility of 68Ga-DOTATOC-PET/MRI for ONSM diagnosis (Figure 2). After an intravenous injection of median 168 MBq (IQR, 154–182) 68Ga-DOTATOC, static images were obtained in seven patients 57 min post-injection (IQR, 50–77; PET acquisition time = 20 min) and dynamic acquisition was performed in one patient over 70 min post-tracer injection. On contrast-enhanced T1-weighted MPRAGE images, seven of ten ONSM were difficult to delineate. Contrarily, nine out of ten ONSM presented with intense positive receptor stocking on 68Ga-DOTATOC-PET. In three of the nine lesions, the diagnosis of WHO grade I ONSM was confirmed histopathologically. The median of the standard uptake value (SUV) max was 5.6 (IQR, 2.6–7.8) and the median SUVmean was 3.5 (IQR, 1.9–5.2). One small lesion (patient 5, right eye, size 4 mm) presented a very low and nearly absent PET signal (SUVmax 1.3).

### 3.3. RRS

We further assessed the role of 68Ga-DOTATOC-PET/MRI in RRS planning. For all nine ONSM that presented 68Ga-DOTATOC uptake, hybrid PET/MR imaging was used for target volume contouring. One ONSM was contoured only on CT and MR images due to the nearly absent PET signal. The median PTV and GTV were 0.83 cm^3^ (IQR, 0.51–2.22) and 0.83 cm^3^ (IQR, 0.32–2.06), respectively. Importantly, for three ONSM (patient 5 (*n* = 2) and patient 8 (*n* = 2)), the PTV and GTV differed because the lesions were difficult to delineate, as patient 5 presented with two very small ONSM (GTV1 = 0.29 cm^3^ and GTV2 = 0.05 cm^3^), and patient 8 presented with diffuse ONSM spread postoperatively with a relatively low PET uptake (GTV 1.44 cm^3^; SUVmax 2.9). All ONSM were treated in five fractions with a median surrounding dose of 25.0 Gy in total (IQR, 22.5–25.0), normalized to an isodose of 70–85%, which resulted in maximal doses between 26.5 and 35.7 Gy. The median maximal dose to the optic pathway was 23.5 Gy; a higher dose was allowed if the corresponding eye was blind (*n* = 3).

### 3.4. Follow-Up

The median follow-up time after RRS was 11.7 months (IQR, 9.4–16.4). All ONSM presented as stable disease. RRS resulted in improved visual function in two of the eight patients (visual acuity before and after RRS, 0.2 vs. 0.8 and 0.6 vs. 0.8), while six patients showed stable vision (Table 1). In the three ONSM patients who were already blind in the affected eye before the start of treatment, no improvement or regeneration of vision after RRS was observed.

### 3.5. Literature Review

According to the described search criteria, all studies on 68Ga-DOTATOC-PET/MRI for meningiomas and 68Ga-DOTATOC-PET/CT for ONSM were included in the literature review. They consisted of one retrospective study, one prospective study, two case reports, and two comparative single-cohort studies. General information, diagnostic predictability, use for radiotherapy planning, and limitations are presented in Table 2.

## 4. Discussion

In our study, the suspected diagnosis was substantiated in 9/10 lesions by positive receptor stocking of Ga68-DOTATOC. In three cases, the diagnosis was confirmed by histopathology. PET information combined with additional MR image information provided a secure basis for successful treatment planning of ONSM. Thus, Ga68-DOTATOC-PET/MRI can be considered a safe one-stop-shop image tool prior to RRS for ONSM.

Radiosurgery has increasingly been applied to ONSM and offers a similar local control with only a few side effects in comparison to other irradiation techniques, as shown in our previous publication [20]. Here, we focus on the feasibility of 68Ga-DOTATOC PET/MRI in radiosurgical treatment of ONSM. For all nine ONSM that presented 68Ga-DOTATOC uptake, hybrid PET/MRI imaging (contrast-enhanced T1-weighted MPRAGE and/or fat-suppressed T1-weightes sequences and attenuation-corrected PET images) was used for PTV contouring. In our opinion, the PET signal provides a good overview of the location of meningiomas, especially in postoperative patients and patients with bilateral tumors or canalicular location. In addition, hybrid imaging of 68Ga-DOTATOC-PET/MRI enables precise contouring (fine-tuning) based on MRI, making it superior to the combination of PET and CT or MRI alone. Overall, PET/MRI enabled the avoidance of uncertainty and the inclusion of additional safety margins for radiation planning in 7/10 lesions. In the other 3/10 cases, the tumors were difficult to delineate because of diffuse spread postoperatively or due to very small size. In patient 5, one of these tumors presented a nearly absent PET signal (SUVmax = 1.3). This fact could be explained by the partial volume effect, as the resolving power is limited. For the Siemens Biograph PET/MRI, studies using PET image quality phantom (Esser) filled with fluorodeoxyglucose (F18) presented a detectability of the spheres down to 5 mm when sphere-to-background activity concentrations were 8:1 and acquisition time was 180 s. The size of the lesion in patient 5 was 4 mm and therefore beneath the resolving power. Furthermore, the smallest resolvable rod diameter differs between the radionuclides and is larger for Ga68 than for F18 [26].

Primary optic nerve tumors in adults can be ONSM, as well as gliomas, lymphomas, leukemic infiltration, or gangliogliomas [27]. The diagnosis of ONSM using MRI alone is not always possible [28]. The most common causes for misdiagnosis are clinician assessment failure, followed by errors in diagnostic testing [29]. Clinical misdiagnoses include papillitis, optic atrophy, ischemic optic neuropathy, and optic neuritis [30,31]. Patients with initial misdiagnosis are not rare, which can lead to delayed treatment initiation with increased risk of irreversible visual deterioration up to blindness. Particularly in cases with unclear diagnosis, hybrid imaging techniques can help to increase the diagnostic accuracy by using somatostatin receptor 2 analogues [10,32,33]. Eckert et al. [34] have already used the additional information of somatostatin-receptor-analogue PET in the majority of their patients to prove the correct diagnosis before the treatment and to better delineate the ONSM for target contouring prior to intensity-modulated radiotherapy. In the study by Klingenstein et al. [7], 10 of 13 symptomatic patients with ambitious lesions of the optic pathway presented increased 68Ga-DOTATATE uptake on PET/CT, indicating the presence of meningiomas. Five tumors (four DOTATATE-positive and one DOTATATE-negative) were histologically examined; four meningiomas and one non-meningioma (inflammatory collagenous connective tissue) were confirmed, in accordance with the PET findings. In this study, 68Ga-DOTATATE-PET/CT demonstrated a sensitivity and specificity of 100%. In our study, 9/10 lesions presented positive receptor stocking. The one case without significant receptor stocking could probably be explained by the partial volume effect due to the small lesion size. Nevertheless, there is no histological evidence, and differential diagnosis cannot be excluded.

In a case report by Feghali et al. [6], 68Ga-DOTATATE-PET/CT imaging was performed in a patient with left temporal hemianopsia and a dull pain in the left eye after inconclusive MRI findings. PET/CT showed asymmetric fusiform enlargement of the left optic nerve with an associated abnormal diffuse radiotracer uptake, which supported the diagnosis of ONSM.

As mentioned above, only a few studies have been published on PET imaging in ONSM [6,7], and so far, no studies based on PET/MR imaging have been published. The use of PET/MRI with the radiotracer 68Ga-DOTATOC is particularly beneficial in sensitive regions, such as the optic nerve canal and brain stem, and generally for less experienced physicians, as it provides improved imaging of small infiltrative regions compared to MRI and CT alone [16,24]. Despite the high lesion-to-background ratio, in cases of adjacent meningioma and pituitary gland with physiologically high somatostatin receptor binding, the distinction between the two remains challenging [19].

Compared with PET/CT, one advantage of PET/MRI is the reduced radiation exposure of the patient. Moreover, MRI is the preferred imaging tool for ONSM compared with CT [5]. Comparative studies showed no significant difference between the lesion volumes delineated from PET/CT and PET/MRI, with a high correlation (*r* = 0.99), even for very small lesions (0.2 cm) [30], suggesting that lesion delineation was driven by the PET component. A limitation of the comparative studies is the sequential image acquisition of PET/CT and PET/MRI after radiotracer injection, which leads to different tracer uptake times in both imaging techniques. To compensate for the delay between PET/CT and PET/MRI, the scanning duration was prolonged in PET/MRI [8,25]. If the scanning time is significantly prolonged, artifacts due to head movement should be considered, and movement correction should be evaluated [25].

Since PET/MRI is already a promising tool for radiotherapy planning in meningiomas [16], its use should also be considered in ONSM. Further large-scale prospective studies, most likely multicenter due to the low incidence of ONSM, could support the current findings by investigating the role of PET/MRI in ONSM diagnosis, treatment planning, and follow-up.

The size of meningioma lesions after radiotherapy is usually described as stable or subtly regressive [35,36,37]. Likewise, none of our patients presented progression in tumor size during the short-term follow-up, but long-term outcomes remain to be evaluated. Using hypofractionated radiosurgery is an effective treatment option for ONSM [15] with long-term tumor control [38,39]. The risk of radiation-induced optic pathologies is rare (<1%) when a maximum dose of less than 25 Gy to the optic pathway is applied in five fractions and increases to 1.9% for 30 Gy [40]. Overall, two of our patients showed improved visual acuity after robotic radiosurgery using CyberKnife, whereas all others presented stable visual acuity.

Our results are generally in concordance with those of previous studies and suggest that 68Ga-DOTATOC-PET/MRI is a secure imaging tool for successful treatment planning in patients with ONSM before treatment with CyberKnife.

### Limitations

This was a retrospective study with a limited number of patients with suspected ONSM. Nevertheless, it should be considered as a proof-of-concept study for 68Ga-DOTATOC-PET/MRI before RRS for ONSM as it provides satisfactory diagnostic results. Additional histological confirmation was available only for a minority of patients. Nevertheless, DOTATOC-PET/MRI is experiencing high clinical acceptance as a surrogate for non-invasive diagnosis, as the tracer is known to have a high sensitivity and specificity for the detection of meningiomas. The follow-up period is relatively short because PET/MRI was only available at our institution since 2016. Moreover, it was used for diagnosis and radiation planning of ONSM before RRS only since 2018. The literature review was dominated by retrospective studies and case reports, except for one prospective study. Therefore, all the results should be viewed with caution and require ongoing research.

## 5. Conclusions

Ga68-DOTATOC-PET/MRI before RRS for ONSM allows safe treatment planning through combined information about spatial spread in MRI and PET. For radiation oncologists, using hybrid imaging may enable the avoidance of uncertainties, including additional safety margins for radiation planning. Due to specific receptor stocking, diagnostic accuracy can be improved. The extent to which the therapeutic outcome improves through a potentially better delineation of the ONSM using PET/MRI compared with MRI or CT alone, must be evaluated in future prospective studies.

## Figures and Tables

**Table 1:** Visual acuity ^1^ per patient pre- and post-treatment.

Pre-Radiosurgery	Post-Radiosurgery
blind	blind
0.2	0.8
0.6	0.8
light/dark	light/dark
1	1
blind	blind
0.9	0.9
0.7	0.7
0.05	0.05
blind	blind

^1^ Visual acuity was objectively measured in decimal scale by patients’ ophthalmologists as required by the European Norm (EN ISO 8596).

**Table 2:** Literature review results regarding somatostatin receptor-based PET/MRI in meningiomas and PET/CT in ONSM.

Author/Study Type	Year	Number of Patients	Tumor Type	Scanner	Diagnostic Predictability and Use for Radiotherapy Planning	Limitations
Acker, G. [16]/ Retrospective study	2019	10	meningiomas	PET/MRI (Biograph mMR, Siemens Healthcare GmbH)	Impact on target volume, contouring of skull-based meningiomas, radiosurgery treatment planning	Small number of patients; lack of standardization for PET data interpretation
Afshar-Oromieh, A. [19]/ Comparative single cohort study	2015	15	meningiomas	PET/MRI (Biograph mMR; Siemens Healthcare GmbH)	Ideal combination of high sensitivity/specificity (PET) and best morphological visualization (MRI); distinction between pituitary gland and meningioma can be difficult	Small number of patients; differences in tracer uptake values in PET/CT and PET/MRI possibly due to different detectors or algorithms of measurement; no histological confirmation of meningioma diagnosis for every lesion
Thorwarth, D. [24]/ Case report	2011	1	meningiomas	APD based PET detector inserted in a Magnetom Trio MRI (Brain PET; Siemens Healthcare)PET/CT (Biograph HiRez 16, Siemens Healthcare GmbH)	Possibly beneficial for target volume definition	Case report; lack of standardization for PET data interpretation; after tracer injection decay between PET/CT and PET/MR image acquisition; compensating prolonged acquisition protocol in PET/MRI
Boss, A. [25]/ Comparative single cohort study	2010	3	meningiomas	APD based PET detector inserted in a Magnetom Trio MRI (BrainPET; Siemens Healthcare GmbH);PET/CT (Hi-Rez Biograph 16; Siemens Healthcare GmbH)	Image quality + quantitative data achieved using PET/MRI similar to PET/CT: meanpaired difference of the tumor volumes was 0.2 × 6 × 1.3 cm^3^; advantages such as higher soft-tissue contrast, reduced radiation exposure, and advanced MRI techniques	Without 4 mm Gaussian filter slight streak artifacts; after tracer injection decay between PET/CT and PET/MR image acquisition
Al Feghali, K. A. [6]/ Case report	2018	1	ONSM	PET/CT (n/a)	Probable, not definitive	Lack of biopsy
Klingenstein, A. [7]/ Prospective study	2015	10	ONSM	PET/CT (Biograph 64 TruePoint; Siemens Healthcare GmbH; Discovery 64-slice; GE Healthcare)	100% specificity; 100% sensitivity	Small number of patients; histological proof only in 5 patients

PET, positron emission tomography; MRI, magnetic resonance imaging; CT, computer tomography; n/a, not available; ONSM, optic nerve sheath meningioma; APD, avalanche photodiode.

## Data Availability

The datasets generated during and/or analyzed during the current study are not publicly available due to the protection of data privacy but are available from the corresponding author upon reasonable request as an anonymous set.
